# A Fatal Case of Tuberculosis Meningitis in Previously Health Children

**DOI:** 10.3390/pediatric14020024

**Published:** 2022-04-09

**Authors:** Manuela Colosimo, Antonella Caruso, Salvatore Nisticò, Pasquale Minchella, Antonio Cutruzzolà, Simona Paola Tiburzi, Virginia Vescio, Filippo Luciani, Gianmarco Marcianò, Luca Gallelli

**Affiliations:** 1 Operative Unit of Microbiology and Virology, Pugliese Ciaccio Hospital, 88100 Catanzaro, Italy; manuelacolosimo@hotmail.it (M.C.); antonella.caruso1988@gmail.com (A.C.); snistico1@virgilio.it (S.N.); pminchella@aocz.it (P.M.); 2 Department of Medical and Surgical Sciences, University “Magna Graecia” of Catanzaro, 88100 Catanzaro, Italy; a.cutruzzola@unicz.it; 3 Operative Unit of Anaesthesiology and Intensive Care, “Pugliese Ciaccio” Hospital, 88100 Catanzaro, Italy; tiburzi1@virgilio.it; 4 Operative Unit of Neurology, “Pugliese Ciaccio” Hospital, 88100 Catanzaro, Italy; virginiavescio@gmail.com; 5 Infectious Diseases Unit of Annunziata Hospital, 87100 Cosenza, Italy; filippo.luciani@gmail.com; 6 Clinical Pharmacology and Pharmacovigilance Unit Mater Domini Hospital, 88100 Catanzaro, Italy; gianmarco.marciano@libero.it; 7 Medifarmagen SRL, Mater Domini Hospital, 88100 Catanzaro, Italy; 8 Research Centre FAS@UMG, Department of Health Sciences, University “Magna Graecia” of Catanzaro, 88100 Catanzaro, Italy

**Keywords:** tuberculous meningitis, young, non-specific symptoms

## Abstract

Tuberculous meningitis (TBM) is a severe form of tuberculosis. We report the development of fatal TBM in a 2-year-old previously healthy child, suggesting that TBM must be evaluated in children of all ages with non-specific symptoms of central nervous involvement because a diagnostic delay induces a negative prognosis.

## 1. Introduction

Tuberculous meningitis (TBM) is a severe form of tuberculosis that induces severe neurologic complications [1]. A Cochrane review, analyzing 18 studies in adults and children with TBM, documented a mortality rate of 30%, and death occurred mainly in 6 months [2].

Tuberculosis infection occurs through the inhalation of aerosol droplets containing *M. tuberculosis bacilli*. *M. tuberculosis* crosses the lung epithelium and activates lung cells (e.g., alveolar macrophages, neutrophils, and dendritic cells), leading to the release of cytokines and chemokines that contribute to immune protection [3].

TBM can develop when *M. tuberculosis* breaches its barrier, inducing the development of parenchymal (cortical), and meningeal tuberculomas [4,5,6].

Both clinical evaluation and microbiological tests represent the first line for TBM diagnosis. In particular, the demonstration of TBM in cerebrospinal fluid (CSF) using smear microscopy, culture, and DNA amplification testing is mandatory [7]. Early diagnosis is required to provide better pharmacological treatment [4].

In 75% of children with TBM, the onset of symptoms takes less than 12 months [8] and it induces very high mortality even if diagnosed and treated. About 20% of children die, and of those surviving over half have neurological disability [9].

In the present case, we describe the development of TBM in a young, previously healthy child.

## 2. Case Report

A 2-year-old girl, weight 10 kg, high 82 cm was transferred to our hospital on 6 June 2021, for fever (38.5 °C), headache, and convulsions. History documented the presence of convulsions in her uncle but excluded other diseases in her parents. Routine tests for Human Immunodeficiency Virus, Citomegalovirus, Epstein–Barr Virus, Rubivirus, Leishmania, Enterovirus in the blood and SARS-CoV-2 in the nasal swab were negative.

A neurological examination documented a score of 10 in the Glasgow Coma Scale (GCS): eye’s response: secondary to sound/verbal stimuli (score: 3); verbal’s response: crying (score: 3); motor’s response: flexion of the limbs (score: 4). Brain magnetic resonance imaging documented a dilatation of the lateral ventriculi with a transependimal resorption of cerebrospinal fluid (CSF) without abnormal leptomemingeal enhancement in the basal cisterns or sylvian fissures.

The CSF examination documented a clear and colorless appearance, with an increase in cell count (162 cells/μL, normal range 0–25 cells/μL), albumin (438 mg/L, normal range 0–350 mg/L), and immunoglobulins (Ig) G (44 mg/L, normal range 0–34 mg/L) (Table 1), with a positivity for herpes virus (HHV-6; sensitivity 10,000 copies/mL) (FILMARRAY™ Meningitis/Encephalitis (ME) Panel, BioMerieux, Grassina(FI), Italy) (Table 2), and for oligoclonal bands with intrathecal synthesis.

Therefore, a treatment with acyclovir (100 mg tid), methylprednisolone (100 mg/day), and Igs (5 gr/day) was started. Furthermore, for the recurrence of seizures, levetiracetam (300 mg/day) was added. One week later (13 June 2021), due to the persistence of fever (38.6 °C) with prolonged focal seizures (about 8 min), characterized by open eyes, fixed gaze, hypertensive neck, stiffening and shaking of the left upper limb, midazolam (1 mg) was started (GCS: 7). Nuclear magnetic resonance of the brain revealed a dilation in the lateral and third ventricles with signs of transependimal CSF resorption and a diffuse area of restriction (i.e., bilateral cerebellar hemispheric sites, bilateral temporomesial, left cerebral peduncle, splenium of the corpus callosum, in the cortex of parietal gyri bilaterally, in the motor and sensory cortex on the right).

Two days later (15 June 2021), the persistence of impaired clinical conditions (GCS: 6) induced a new evaluation in her parents, and her mother declared that for one year she had suffered from untreated active tuberculous pneumonia. History revealed that her mother, a single woman, lives with two children of Romanian ethnicity in poor economic and health conditions. The child has not recieved BCG (Bacille Calmette-Guérin) or other vaccinations during childhood, although repeatedly recalled by the social services.

Therefore, both the Mantoux test and the Quantiferon dosage were quickly performed on the young patient, with negative results, but on 17 June 2021, CSF was positive for TBM (GeneXpert MTB/RIF ultra-assay, Cepheid, Buccinasco (MI), Italy) (Table 1).

Direct microscopic examination at 40X following auramine staining (TB Auramine M Stain, Becton Dickinson, Milan, Italy) revealed the presence of tuberculous bacilli acid-resistant alcohol (BAAR), with a title (++). The culture was performed on liquid medium (BBl MGIT Becton Dickinson) and Lowenstein-Jensen solid medium (Becton Dickinson), highlighting BAARs growth. Using the reverse hybridization system (GenoType MTBC, Hain Lifescience, Nehren, Germany), colonies were identified as Mycobacterium tuberculosis. Mycobacterial antibiogram (SIRE kit and PZA kit, Becton Dickinson) showed a high sensibility to all antibiotics tested (i.e., ethambutol, isoniazid, pyrazinamide, and rifampicin).

Other biological samples (i.e., gastric fluid, bronchoalveolar lavage, and urine) were tested to evaluate the diffusion of mycobacterial infection, with negative results. In contrast, bronchoalveolar lavage was positive for *P. aeruginosa* and *A. baumanii*, while gastric fluid and catheter were positive for *C. guilliermondii*, and blood culture for *C. parapsilosis*. Species identification was carried out using the MALDI coupled to time-of-flight mass spectrometry (MALDI-TOF-MS; Biomerieux, Craponne, France) and the relative antimicrobial sensitivity/resistance using Vitek 2 (Biomerieux) and MicroScan WalkAway plus System (Beckman Coulter, Italy).

**Figure 1:**
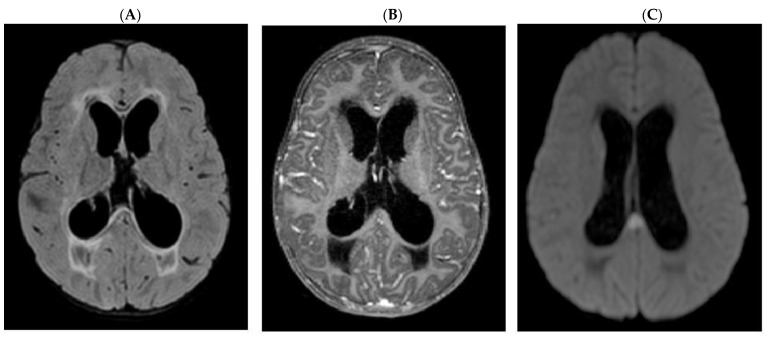
Brain magnetic resonance: (**A**) FLAIR axial scans showing hydrocephalic dilatation of the lateral ventricles and the third ventricle with marked transependimal resorption of CSF; in T1 TSE, absence of contrast enhancement (**B**). (**C**) in DWI axial scans ischemic lesions in the splenium of the corpus callosum.

Brain computer tomography (CT) scans showed an increase in brain damage (GCS: 5). It was confirmed by magnetic resonance imaging that showed an increase in brain damage (Figure 1): increased hydrocephalus and transependimal resorption of CSF (A), increased ischemic lesions with involvement of the cerebellar hemispheres, cerebral cortex, midbrain (not shown) and splenium of the corpus callosum (C). However, no contrast enhancement is visible in T1-weighted images (B).

A poly-therapy with pyrazinamide (150 mg/day), rifampicin (160 mg/day), isoniazid (150 mg/day), and ethambutol (150 mg/day) was started without clinical improvement. The child was transferred to the intensive care unit, where, after intubation and sedation (midazolam 1 mg/day), an external ventricular lead (EVD) was placed and a treatment with pantoprazole (10 mg/day), dexamethasone (2 mg/day), carbamazepine (7.5 mL bid), levetiracetam (100 mg/day) and enoxaparin sodium (1000 IU) was administered.

On 6 July 2021, due to the impairment of clinical condition (development of respiratory failure; blood pressure oxygen of 82%) (GCS: 3), a ventricle peritoneal shunt was placed, and a tracheostomy was performed with a rapid improvement in clinical conditions. The pupil diameter improved, and the motor response appeared after painful stimulation (internal rotation of the shoulder girdle with a hint of rotation of the right upper limb). More lively movements in the right lower limb, and the presence of the sucking reflex were appreciated (GCS: 5).

On 12 July 2021, thorax X-ray and bronchoscopy culture examination revealed a respiratory infection sustained by *P. aeruginosa* (MALDI-TOF system, Biomerieux, France; Vitek 2, Biomerieux; MicroScan WalkAway plus System, Beckman Coulter, Italy). Therefore, meropenem (400 mg/day initial dose and then 200 mg every 8 h) was administered. On 22 July 2021, culture tests showed positivity for *C. guilliermondii* and mycamine (20 mg/day) was added. CSF documented a positivity for *M. tuberculosis* as follows: (162 cells/μL, 0–25 cells/μL normal range) albumin (438 mg/L, normal range 0–350 mg/L) and IgG (44 mg/L, normal range 0–34 mg/L). The microscopic examination following auramine staining revealed the presence of BAAR, title (+−).

On 1 September 2021, the clinical conditions were impaired (GCS: 3). A culture examination of bronchoaspirate was positive for *A. baumanii,* while blood cultures for *C. parapsilosis*. Both the microorganisms were unresponsive to antimicrobial infection. About 10 days later, the child died.

## 3. Discussion

We reported a case of a fatal TBM in a young, previously healthy child. It has been reported that *M. tuberculosis* can cause bacterial meningitis, alongside pathogens such as *N. meningitidis*, *H. influenzae*, or *S. pneumoniae* [10,11]. Approximately one-half of all TBM infections lead to severe disability or death [12].

The occurrence of TBM is rare in children who are less than three months of age [13], and the TBM peak incidence in children is between 2 and 4 years of age [14].

In agreement, we reported the TBM’s development in a 2-year-old girl without pulmonary symptoms or previous systemic diseases. The history, symptoms, and CSF suggested an infection sustained by HHV-6, resulting in the diagnostic delay.

HHV-6 is a microbiological infection sustained by human herpesvirus 6 and characterized by benign febrile illness with or without skin rash (roseola), otitis, and diarrhea [15]. It might also cause several diseases of the central nervous system (e.g., encephalitis). A brain magnetic resonance imaging study shows a hippocampal abnormality and pontine lesions. In a recent study, Chencheri et al. [16] reported the development of HHV-6 in children up to 2-years-old. In these patients, the CSF analysis showed positive PCR for HHV-6, normal glucose levels (80 mg/dL; normal range 50–80), and protein levels (16 mg/dL; normal range 15–45), and a prevalence of lymphocytes (normal 2 mononucleates).

The clinical onset of TBM may be acute, subacute, or gradual [17] and is characterized by non-specific symptoms in the early stages, such as malaise, low-grade fever, symptoms related to pulmonary TB, and/or flu-like illness [18]. Children with more advanced disease may have signs of meningeal irritation, cranial nerve palsies, neurological deficits, altered sensorium, and movement disorders [14]. In these patients, CSF analysis shows low glucose, high protein, and a predominance of lymphocytes.

The early differential diagnosis between HHV-6 and TBM could be performed with an accurate history and with CSF data. Unfortunately, the low social status and the distrust of the mother of Romanian ethnicity delayed the diagnosis.

In fact, even if brain magnetic resonance was not suggestive of an infection, the analysis of CSF revealed the presence of HHV-6 with normal glucose and high protein; therefore, a diagnosis of HHV-6 was performed, and the treatment was started.

We re-reviewed the parents’ history after the failure of antiviral treatment, suggesting that the presence of HHV-6 in CSF has uncertain clinical significance.

In the present case, symptoms were subacute, and only a new clinical evaluation allowed us to identify the presence of active pulmonary TB in her mother, suggesting the research of *M. tuberculosis* in the CSF of the child.

This point could be of interest because the mother of the child, probably out of distrust or other social stigma, revealed the TB infection when the child’s clinical condition deteriorated, suggesting that she did not consider this information to be relevant.

The positivity to MT induced the start of a specific but delayed treatment with the death of the child.

The TB’s negativity in CSF in the first sample (admission) may be related to the following different points: (i) 10 mL of CSF are recommended for the grown of MT and for its diagnosis; (ii) TBM is a paucibacillary disease such that no diagnostic test (including Xpert MTB/Rif ULTRA) has adequate sensitivity to rule out the disease when negative; (iii) cultures take up to six weeks for positive identification [19].

A systematic review of 19 pediatric TBM studies, including a total of 1636 children with TBM, reported a mortality rate of 19%, with a 54% risk of neurologic complications among survivors. [20]. Mortality is higher in premature childhood, while young children are at a higher risk of progression to severe forms of TB [21].

In conclusion, this case report suggests that TBM must be evaluated in children of all ages with non-specific symptoms of central nervous involvement. Therefore, Xpert MTB/Rif could perform an early diagnosis to start an earlier treatment that represents the most important predictor of survival.

## Figures and Tables

**Table 1:** Time course of cerebrospinal fluid (CSF) characteristics of the child examined during the study.

	6 June	15 June	Normal Range
Appearance	Clear, colorless	Clear, colorless	
Glucose	67	68	45–80 mg/dL
Cells count	162	170	0–25 cells/μL
Polymorphonuclear cells	34	29	0–20 per mm^3^
Monocyte cells	128	141	0–20 per mm^3^
Albumin	438	450	0–350 mg/L
IgG	44	50	0–34 mg/L

**Table 2:** Pathogens evaluated through PCR-Filmarray (BioMerieux, Italy).

Bacteria	Virus	Yeasy
Escherichia coli K1, Haemophilus influenzae, Listeria monocytogenes, Neisseria meningitidis, Streptococcus agalactiae, Streptococcus pneumoniae	Cytomegalovirus, Enterovirus, Herpes simplex virus 1, Herpes simplex virus 2, Human herpes virus 6, Human parechovirus, Varicella zoster virus	Cryptococcus neoformans/gattii.

## Data Availability

Not applicable.
